# Clinical Characteristics and Outcomes of Invasive *Listeria Monocytogenes* Infection: A 45-Year Retrospective Study at a US Tertiary Center

**DOI:** 10.1093/ofid/ofag496

**Published:** 2026-08-12

**Authors:** Ayesha Samreen, Maria Alejandra Mendoza, James J Vaillant, Omar Abu Saleh

**Affiliations:** Division of Public Health, Infectious Diseases, and Occupational Medicine; Mayo Clinic, Rochester, Minnesota, USA; Division of Infectious Diseases, University of Utah, Salt Lake City, Utah, USA; Department of Internal Medicine, Section of Infectious Diseases, Marshall University Joan C. Edwards School of Medicine, Huntington, West Virginia, USA; Division of Public Health, Infectious Diseases, and Occupational Medicine; Mayo Clinic, Rochester, Minnesota, USA

**Keywords:** gentamicin therapy, invasive listeriosis, mortality predictors

## Abstract

**Background:**

Invasive listeriosis remains a leading cause of foodborne infection–related mortality in the United States and is associated with substantial case fatality despite appropriate antimicrobial therapy. Owing to the relative rarity of invasive disease, contemporary data evaluating clinical characteristics, optimal antimicrobial strategies, and predictors of mortality remain limited.

**Methods:**

We conducted a retrospective cohort study of adults with laboratory-confirmed invasive *Listeria monocytogenes* infection at Mayo Clinic Health System sites from 1980 to 2024. Clinical syndromes were categorized into 4 mutually exclusive categories, namely bloodstream infection, central nervous system infection, pregnancy-associated infection, and other focal infections. The primary outcome was 1-year all-cause mortality. Secondary outcomes included 90-day mortality and residual neurological deficits among patients with neurolisteriosis.

**Results:**

Fifty patients were included (median age, 72 years; 56% immunocompromised). The most common clinical presentations were bacteremia (44%) and neurolisteriosis (36%), followed by endovascular infection (12%) and peritonitis (6%). Most patients received a beta-lactam–based regimen, 46% aminopenicillin monotherapy, and 30% received combination therapy with gentamicin. Thirty-day, 90-day, and 1-year mortality were 10%, 12%, and 20%, respectively. Immunocompromised status and intensive care unit admission were associated with an increased risk of 1-year mortality, whereas adjunctive gentamicin therapy was not associated with improved survival.

**Conclusions:**

Invasive listeriosis is associated with substantial morbidity and mortality, particularly among immunocompromised hosts. Host factors and severity of illness at presentation appear to be key drivers of unfavorable outcomes. Larger multicenter studies are needed to define optimal management strategies.

## Graphical Abstract

**Graphical Abstract:**
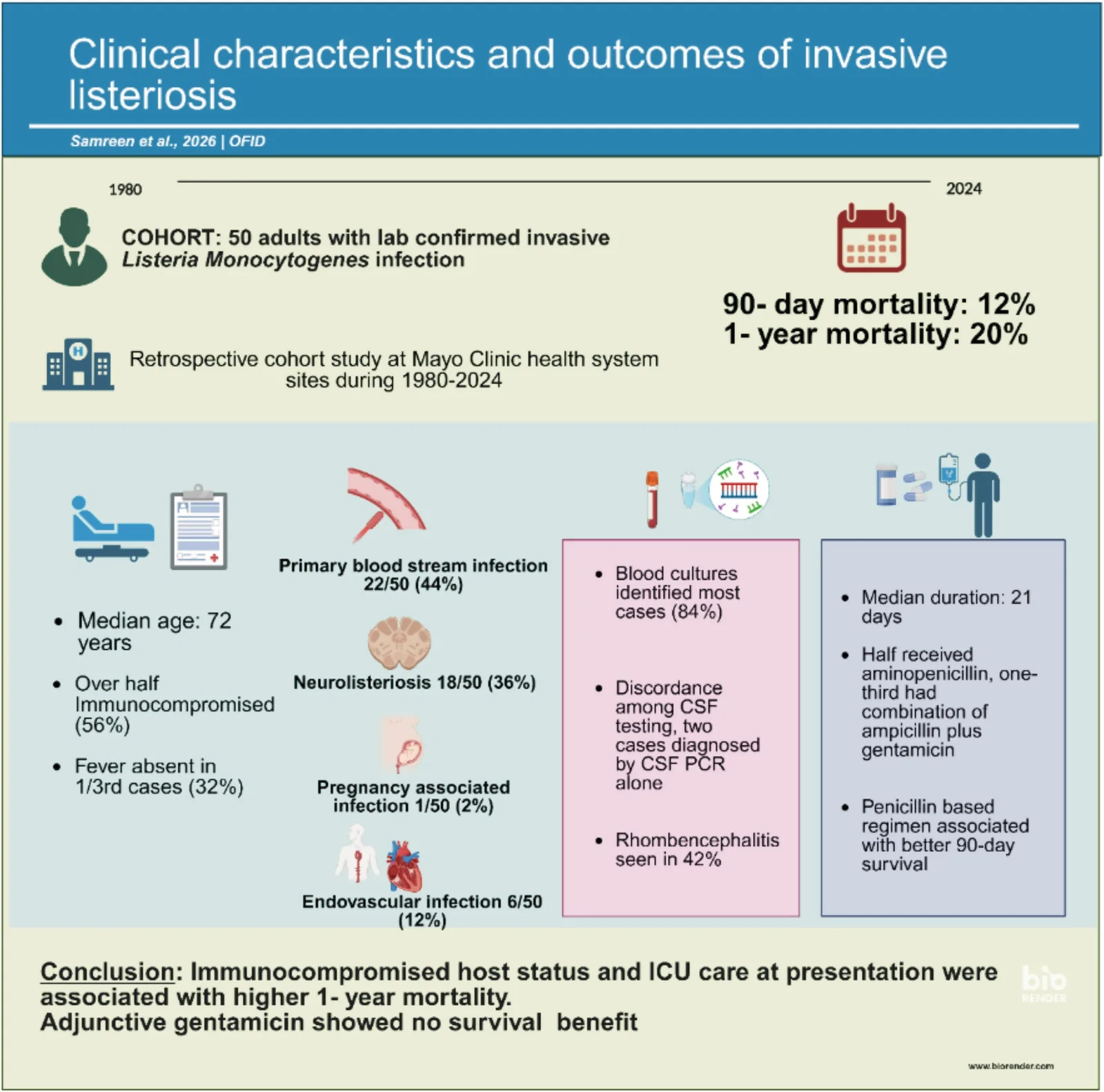
Graphical summary of clinical presentation, management, and outcomes of invasive listeriosis. Created in BioRender. samreen, A. (2026) [24]

*Listeria monocytogenes* is a foodborne pathogen responsible for approximately 1600 cases of invasive infection annually in the United States and remains associated with a mortality rate of 20%–30% despite appropriate antimicrobial therapy [1–4]. Invasive disease most commonly affects older adults, pregnant individuals, and immunocompromised hosts and typically manifests as bacteremia, central nervous system infection, or pregnancy-associated infection [5, 6].

Ampicillin is considered first-line therapy, sometimes in combination with an aminoglycoside such as gentamicin for synergy, although this remains debated [2, 5–8]. Alternative agents including trimethoprim-sulfamethoxazole, vancomycin, or fluoroquinolones are used in cases of intolerance, and the role of adjunctive steroids in neurolisteriosis remains uncertain [9–11].

Despite insights from large European cohorts (eg, MONALISA and Danish cohorts) [12, 13], contemporary data evaluating predictors of short- and long-term mortality, as well as the impact of adjunctive antimicrobial therapy, remain limited—particularly in the United States. We therefore conducted a 45-year retrospective study of adult patients with invasive *Listeria monocytogenes* at a large tertiary referral center in the United States to characterize clinical syndromes, management patterns, and predictors of mortality, with a focus on long-term outcomes and the role of adjunctive gentamicin therapy.

## METHODS

### Study Design and Setting

We conducted a retrospective multicenter cohort study across Mayo Clinic Health System Sites from 1 January 1980, to 31 December 2024, including adult patients with laboratory-confirmed invasive *L. monocytogenes* infection. Cases were identified through microbiology laboratory records. Invasive infection was defined as isolation of *L. monocytogenes* from a normally sterile site, by culture or molecular methods, including polymerase chain reaction (PCR) from blood, cerebrospinal fluid (CSF), peritoneal fluid, or operative specimens.

### Data Collection

Clinical data were abstracted from electronic and archived medical records. Collected variables included demographics, comorbidities, immunocompromised status, clinical presentation, exposure history, diagnostic findings, antimicrobial therapy, and outcomes. Patients were categorized into 4 mutually exclusive clinical syndromes based on their predominant presentation, when overlapping syndromes were present: (1) primary bloodstream infection without an identifiable focus, (2) central nervous system infection (neurolisteriosis), (3) pregnancy-associated infection, and (4) other focal infections, including endovascular infection and peritonitis.

### Case Definitions

- **Primary bloodstream infection:** Isolation of *L. monocytogenes* from blood cultures without an identifiable focal source and in the absence of clinical or radiographic evidence of CNS, endovascular, intra-abdominal, or pregnancy-associated infection.
- **Central nervous system (CNS) infection (neurolisteriosis):** Compatible clinical features (eg, fever, altered mental status, nuchal rigidity, or focal neurologic deficits) with either positive CSF culture or molecular test (eg, FilmArray Meningitis/Encephalitis Panel/multiplex PCR) or positive blood culture.
- **Endovascular infection:** Definite or probable native or prosthetic valve endocarditis or vascular graft infection, supported by echocardiography (transthoracic or transesophageal), advanced imaging (eg, PET/CT), intraoperative findings, or persistent bacteremia (>48 hours) in the presence of a cardiac prosthetic device.
- **Peritoneal infection:** Clinical features (abdominal pain or tenderness) and/or radiologic evidence of peritoneal inflammation (eg, imaging showing ascites or peritoneal thickening) with supportive peritoneal fluid findings (WBC >250 cells/µL with neutrophil predominance), and either positive peritoneal fluid cultures or blood cultures for *L. monocytogenes*.
- **Pregnancy-associated infection:** Invasive *L. monocytogenes* infection occurring in a pregnant adult (≥18 years) during pregnancy or index hospitalization. This definition differs from the Council of State and Territorial Epidemiologists surveillance case definition, which includes neonatal listeriosis up to 28 days postpartum. Neonatal cases were excluded as the cohort was restricted to adult patients [14].

In case of overlapping syndromes, classification was based on predominant clinical syndrome guiding management.

Immunocompromised host status was defined as the presence of solid organ or hematologic malignancy, autoimmune disease requiring immunosuppressive therapy or chronic steroid use, solid organ or hematological transplant, functional or anatomical asplenia, or human immunodeficiency virus (HIV) infection. Diabetes mellitus and chronic kidney disease were recorded as comorbidities or risk factors and were not included in this definition.

### Outcomes

The primary outcome was 1-year all-cause mortality, defined as death from any cause within 1 year of diagnosis. Secondary outcomes included 90-day mortality and residual neurological deficits among patients with neurolisteriosis. Residual neurological deficits were defined as new or worsening neurologic abnormalities attributable to the infection, such as altered mental status, gait disturbances, seizures, or cranial nerve deficits that were present upon hospital dismissal.

#### Statistical Analysis

Continuous variables were summarized using medians and interquartile ranges (IQR), and categorical variables as counts and percentages. Comparisons between groups were performed using the chi-square or Fisher's exact test for categorical variables and the Kruskal–Wallis test for continuous variables, as appropriate.

Time-to-event analyses for mortality were performed using Cox proportional hazards regression. Due to the limited number of events, only univariate analyses were performed to avoid model overfitting. Treatment variables were analyzed using logistic regression due to sparse data. A 2-sided *P* value <.05 was considered statistically significant. Statistical analyses were performed using BlueSky Statistics (Version 10.x) [15].

### Ethics Statement

The study was approved by the Mayo Clinic Institutional Review Board with a waiver of informed consent.

## RESULTS

### Patient Characteristics

A total of 50 patients with laboratory-confirmed invasive *L. monocytogenes* infection were included. The median age at diagnosis was 72 years (IQR, 58.3–78.8), and 26 (52%) were male. Twenty-eight patients (56%) were immunocompromised (Table 1). Primary bloodstream infection was the most common presentation (n = 22, 44%), followed by CNS infection (n = 18, 36%), endovascular infection (n = 6, 12%), peritonitis (n = 3, 6%), and pregnancy-associated infection (n = 1, 2%). Clinical characteristics and outcomes of nonpregnancy listeriosis stratified by syndrome are summarized in Table 2.

**Table 1.** Baseline Characteristics of Patients With Invasive Listeriosis

Variable	Patients (n = 50)
Gender (Male)	26 (52%)
Age, years, median (IQR)	72 (58.3–78.8)
Immunocompromised	28 (56%)
Solid organ malignancy	7 (25%)
Hematologic malignancy	7 (25%)
Solid organ transplant	6 (21%)
Autoimmune conditions requiring immunosuppression	10 (36%)
Bone marrow transplant	2 (7%)
Comorbidities	…
Diabetes mellitus	12 (24%)
Chronic kidney disease	12 (24%)
Symptoms on presentation	
Fever	31 (62%)
Fatigue	20 (40%)
Altered mental status	12 (24%)
Diarrhea	12 (24%)
Abdominal pain	11 (22%)
Nausea or vomiting	10 (20%)
Headache	9 (18%)
Clinical syndrome	…
Primary bloodstream infection	22 (44%)
Neurolisteriosis (CNS infection)	18 (36%)
Endovascular infection	6 (12%)
Peritonitis	3 (6%)
Pregnancy-associated infection	1 (2%)
Intensive care unit (ICU) care within 24 h of admission	17 (34%)
Infectious disease consult	43 (86%)
Definitive antibiotic therapy	…
Duration of therapy (Median, IQR)	21 d (14–28)
Ampicillin/Amoxicillin/penicillin alone	23 (46%)
Ampicillin plus gentamicin	15 (30%)
Ampicillin plus Trimethoprim-sulfamethoxazole (TMP-SMX)	7 (14%)
Other regimen (Trimethoprim-sulfamethoxazole, levofloxacin, meropenem, Ampicillin plus ceftriaxone)	5 (10%)
Steroid use (either for critical illness/shock or continuation of preexisting therapy)	14 (28%)
Outcomes	…
Residual neurological deficits	8/18 (44.4%)
Death within 30 d of diagnosis	5 (10%)
Death within 90 d of diagnosis	6 (12%)
Death within 1 y of diagnosis	10 (20%)

Data are presented as number (%) unless otherwise specified. Continuous variables are reported as median (interquartile range [IQR]). Immunocompromised categories are not mutually exclusive, and patients may belong to more than 1 subgroup. Abbreviations: ICU, intensive care unit; TMP-SMX, trimethoprim-sulfamethoxazole.

**Table 2.** Clinical Characteristics and Outcomes of Non–Pregnancy-associated Invasive Listeriosis Stratified by Clinical syndrome

Variable	Primary Bloodstream Infection (n = 22)	Neurolisteriosis (n = 18)	Endovascular Infection (n = 6)	Peritonitis (n = 3)
Median age, years (IQR)	76 (64–82.5)	61 (44.5–69.5)	79 (76.5–79)	62 (61.5–69)
Male sex, n (%)	9 (41%)	9 (50%)	6 (100%)	2 (67%)
Immunocompromised, n (%)	13 (59%)	11 (61%)	2 (33%)	3 (100%)
ICU admission, n (%)	4 (18%)	9 (50%)	3 (50%)	2 (67%)
Median duration of therapy, days (IQR)	14 (14–21)	21 (21–28)	42 (42–42)	28 (14–56)
Aminopenicillin-based therapy, n (%)	15 (68%)	8 (44%)	4 (67%)	2 (67%)
Adjunctive gentamicin use, n (%)	5 (23%)	6 (33%)	1 (17%)	1 (33%)
Other therapies (Meropenem, levofloxacin, TMP-SMX)	2 (9%)	4 (22%)	1 (17%)	0 (0%)
1-year all-cause mortality within subgroup, n (%)	5 (23%)	3 (17%)	0 (0%)	2 (67%)

Data are presented as number (%) unless otherwise specified. Continuous variables are reported as median (IQR). Patients were assigned a single primary clinical syndrome and a single definitive treatment category. Treatment categories were mutually exclusive and defined as aminopenicillin-based therapy (ampicillin, amoxicillin, or penicillin G), aminopenicillin plus adjunctive gentamicin (any exposure during definitive therapy), and other regimens (including TMP-SMX, meropenem, or fluoroquinolones). Abbreviations: ICU, intensive care unit; TMP-SMX, trimethoprim-sulfamethoxazole.

### Clinical Characteristics and Microbiologic Diagnosis

At presentation, fever was present in only 31 (62%) patients, and 17 (34%) required intensive care unit (ICU) admission within 24 hours of presentation. Infectious diseases consultation was obtained in 43 (86%) patients. Exposure history was identified in 27 (52%), most commonly involving high-risk food consumption, including undercooked or processed meats (n = 7) and unpasteurized dairy (n = 4) (Supplementary Table 1). The median interval from symptom onset to hospital presentation was 2.5 days (IQR, 0–6.75), while the median interval from symptom onset to microbiologic diagnosis was 4.0 days (IQR, 2–9). Blood cultures were positive in 38 of 45 patients (84%) in whom they were obtained.

Among the 18 patients with neurolisteriosis, CSF cultures were performed in 17 and were positive in 8 (47%), while CSF PCR was performed in 8 and was positive in 5 (63%). Fourteen patients underwent both CSF and blood cultures; in this subgroup, blood cultures were positive in 10 (71%), including 6 of 8 (75%) patients with negative CSF cultures. Among the 8 patients who underwent both CSF PCR and culture, results were discordant in 4 (3 PCR-positive/culture-negative and 1 PCR-negative/culture-positive), while 2 had concordant positive results and 2 had concordant negative results. Two additional patients were diagnosed by CSF PCR alone despite negative CSF and blood cultures.

Cerebrospinal fluid analysis demonstrated a median protein of 162 mg/dL (IQR, 106.0–286.5), median glucose of 42 mg/dL (IQR, 29.8–81.3), and median white blood cell count of 322.5 cells/mm^3^ (IQR, 118.3–1643.5). Among patients who underwent imaging (n = 13), rhombencephalitis was identified in 5 (39%) (Supplementary Table 2).

Regarding focal syndromes, 6 patients (12%) had endovascular infections, including infective endocarditis (n = 3) and vascular graft infection (n = 3), all occurring in patients over 70 years of age, frequently in the setting of prosthetic cardiovascular material (5/6) or prior aortic aneurysm repair (4/6) (Table 3). Three patients had peritonitis (6%), all were immunocompromised and had positive *L. monocytogenes* peritoneal fluid cultures (3/3). Two pregnant patients in the third trimester were identified: one had placental infection confirmed by culture, and one had meningitis confirmed by CSF PCR and was classified as neurolisteriosis (Supplementary Table 3).

**Table 3.** Endovascular Listeriosis: Clinical Features, Management, and Outcomes

Age	Comorbidities	Presentation	Diagnostic Findings (Imaging/intra Operative)	Final Clinical Diagnosis	Treatment	All-cause Mortality During Follow-up
**79**	DM, CKD, prior AAA repair	Back and abdominal pain	Intraoperative aortic graft cultures positive	Aortic endograft infection + L3–4 discitis	IV ampicillin + TMP-SMX (6 wk), graft explantation, lifelong amoxicillin suppression	No
**78**	Mechanical AVR, pacemaker, Ascending aortic repair	Gastroenteritis, bacteremia	TEE negative	High-grade bacteremia, possible endocarditis	IV ampicillin (6 wk)	No
**79**	TAVR, pacemaker, rheumatoid arthritis on steroids and tocilizumab	Fever	TEE: mitral vegetation	Native mitral valve endocarditis	IV ampicillin + ceftriaxone (6 wk); suppressive therapy with amoxicillin	No
**71**	Rheumatoid arthritis, ILD	Fever, fatigue	TEE: right atrial echo density	Probable endocarditis	IV Penicillin + TMP-SMX (4 wk) followed by TMP-SMX for 2 wk	No
**76**	DM, CKD, prior AAA repair with graft	Back pain, failure to thrive, consumption of unpasteurized dairy	PET/CT: graft FDG avidity, TEE negative	Aortic graft infection	IV ampicillin (6 wk); lifelong suppression with TMP-SMX	Yes
**83**	CKD, DM Prior AVR, AAA s/p repair	Dyspnea, diarrhea	PET/CT: multifocal aortic FDG uptake	Multifocal aortitis with graft involvement	IV ampicillin (6 wk); lifelong suppression with amoxicillin	No

Abbreviations: AAA, abdominal aortic aneurysm AVR, aortic valve replacement; CKD, chronic kidney disease; DM, diabetes mellitus; FDG, 18F-fluorodeoxyglucose; ILD, interstitial lung disease; PET/CT, positron emission tomography/computed tomography; TAVR, transcatheter aortic valve replacement; TEE, transesophageal echocardiography; TEE, transesophageal echocardiography; TMP-SMX, trimethoprim-sulfamethoxazole. Follow-up duration varied across patients. Treatment reflects definitive antimicrobial therapy, including acute therapy, surgical management, and suppressive therapy when applicable.

### Treatment

Most patients received a beta-lactam–based regimen as part of definitive therapy (n = 47, 94%). Aminopenicillin monotherapy was administered in 23 patients (46%), while 15 (30%) received combination therapy with ampicillin and gentamicin. Other regimens included ampicillin plus trimethoprim-sulfamethoxazole (14%), meropenem-based therapy (4%), and alternative agents in cases of intolerance (Table 1). No resistance to ampicillin or trimethoprim-sulfamethoxazole was identified among tested isolates.

The median duration of therapy was 21 days [IQR, 21–28]. Treatment duration varied by syndrome, with longer courses observed in endovascular infection (median, 42 days [IQR, 42–42]) and CNS infection (median, 21 days [IQR, 21–28]) (Table 2).

Corticosteroids were administered primarily for management of septic shock or continuation of preexisting therapy in 28% of patients (n = 14) and were associated with numerically higher hazards of 90-day mortality (HR 2.76; 95% CI .55–13.6; *P* = .214) and 1-year mortality (HR 3.03; 95% CI .76–12.17; *P* = .118), although these associations did not reach statistical significance.

In Kaplan–Meier analysis, patients treated with penicillin-based regimens (including ampicillin, amoxicillin, and penicillin derivatives) demonstrated significantly improved 90-day survival compared with those not treated with penicillin-based regimens (log-rank *P* = .0275) (Supplementary Figure 1).

### Mortality and Outcomes

Thirty-day and 90-day mortality were 10% (n = 5) and 12% (n = 6), respectively. One-year mortality was 20% (n = 10), and this was significantly higher among immunocompromised patients compared with immunocompetent patients (32.1% vs 4.5%, *P* = .015). A small subset of patients (n = 5, 10%) had no identifiable traditional risk factors for listeriosis (age less than 65, nonpregnant, nonimmunocompromised, no diabetes or chronic kidney disease); these patients were aged 52–60 years, presented with CNS disease in 3/5 cases and bloodstream infection in 2/5 cases, and had no deaths at 90 days (0/5). When stratified by decade of diagnosis, overall mortality varied across time periods: 0% (0/1) in 1980–1989, 71.4% (5/7) in 1990–1999, 50.0% (3/6) in 2000–2009, 60.0% (6/10) in 2010–2019, and 23.1% (6/26) in 2020–2024. No consistent temporal trend in overall mortality was observed across decades. In univariable Cox regression analysis for 90-day mortality, decade of diagnosis was not associated with mortality (HR 1.63 per decade, 95% CI .64–4.18; *P* = .31), although estimates were limited by the small number of patients within each time period. Older age (HR 1.09; 95% CI .99–1.20; *P* = .078) and CNS infection (HR 7.86; 95% CI .85–72.99; *P* = .069) showed trends toward higher mortality but did not reach statistical significance.

For 1-year mortality, immunocompromised status (HR 9.54; 95% CI 1.07–84.95; *P* = .043) and ICU admission at presentation (HR 4.65; 95% CI 1.10–19.71; *P* = .037) were associated with increased risk of death. In univariate analysis, inclusion of gentamicin in the treatment regimen was not associated with 90-day attributable mortality (HR 0.40, 95% CI .05–3.42; *P* = .40) (Table 4).

**Table 4.** Univariable Cox Proportional Hazard Analysis of Factors Associated With 90-Day and 1-year mortality

Variable	90-Day Mortality HR (95% CI)	*P* Value	1-Year Mortality HR (95% CI)	*P* Value
Age at diagnosis (per year)	1.09 (0.99–1.20)	0.078	1.04 (0.99–1.09)	.165
CNS infection	7.86 (0.85–72.99)	0.069	Not included	Not included
Male sex	2.33 (0.32–16.8)	0.398	1.79 (0.38–5.88)	.558
**Immunocompromised status**	2.88 (0.32–16.8)	0.377	**9.54** (**1.07–84.95)**	.**043**
**ICU care within 24 h of admission**	2.69 (0.423–10.2)	0.377	**4.65** (**1.10–19.71)**	.**037**

Hazard ratios (HRs) with 95% confidence intervals (CIs) were estimated using univariable Cox proportional hazards regression. Multivariable analysis was not performed due to limited number of events. A 2-sided *P* value <.05 was considered statistically significant. Bold/italicized values indicate statistically significant associations with 1-year mortality (*P* < 0.05). Variables not included in the 1-year mortality model are indicated as “Not included.”

Among 18 patients with neurolisteriosis, 44% (n = 8) developed residual neurological sequelae. In this subgroup, 90-day mortality among patients with positive CSF cultures was 0% (0/7), compared with 33.3% (2/6) in those with negative CSF cultures. In univariate analysis, ICU admission was associated with increased odds of residual neurological deficits (OR 18.9; 95% CI 1.92–650.3; *P* = .031). Gentamicin use was not associated with mortality or neurological sequelae in this subgroup (Supplementary Table 4).

## DISCUSSION

In this single-center cohort spanning 4 decades, invasive listeriosis was associated with substantial mortality and morbidity, particularly among immunocompromised patients and those requiring ICU-level care at presentation. These findings are consistent with prior observational cohorts, including the MONALISA study that identified immunosuppression and neurologic involvement as key determinants of poor outcomes [12, 16]. In our cohort, mortality remained substantial and appeared to be driven primarily by host factors and severity of illness at presentation rather than antimicrobial regimen. Immunocompromised status and ICU admission were the strongest predictors of 1-year mortality, while adjunctive gentamicin therapy was not associated with improved outcomes.

Invasive listeriosis lacks a characteristic clinical presentation and often manifests with nonspecific symptoms that can delay recognition. Fever was absent in over one-third of our patients, consistent with prior studies, which may contribute to delayed diagnosis [17]. A minority of patients in our cohort presented without traditional risk factors, consistent with prior studies demonstrating that listeriosis, including neurolisteriosis, can occur in individuals without classic predisposing conditions [12, 18]. This suggests that host susceptibility may not be fully captured by conventional risk stratification, and that clinical suspicion should remain high in compatible syndromes. Microbiologic diagnosis of CNS disease remains challenging and often requires a multimodal approach. In our cohort, blood cultures identified most cases of neurolisteriosis, including in patients with negative CSF cultures (6 of 8). Additionally, 2 patients were diagnosed only by CSF PCR despite negative cultures, supporting a complementary role for molecular diagnostics in this setting [19–21]. Prior studies have similarly demonstrated that CSF PCR can detect *L. monocytogenes* in culture-negative cases, particularly in the setting of prior antimicrobial exposure, thereby improving diagnostic sensitivity compared with culture alone [20]. *Listeria monocytogenes* is intrinsically resistant to cephalosporins; therefore, commonly used empiric meningitis regimens may not provide adequate coverage. Delayed recognition may contribute to delays in initiation of appropriate targeted therapy [22] Treatment of invasive listeriosis remains an area of ongoing uncertainty, particularly regarding the role of adjunctive aminoglycoside therapy. Most patients in our cohort received beta-lactam–based regimens, and no resistance to aminopenicillins or trimethoprim-sulfamethoxazole was observed, consistent with prior reports [5]. Although unadjusted analyses suggested improved short-term survival among patients treated with penicillin-based regimens, these findings should be interpreted with caution given the exploratory nature of the analysis and small sample size. Across contemporary studies, neurolisteriosis remains associated with substantial mortality, typically ranging from 20% to 40%, and is frequently complicated by persistent neurological deficits among survivors. While the MONALISA study and recent analyses by Sutter et al suggest a potential survival benefit with aminoglycoside-containing regimens, others have not demonstrated consistent benefit [2, 6–8, 10, 12, 16, 17, 23] (Supplementary Table 5). In our cohort, adjunctive gentamicin was not associated with improved survival or neurological outcomes, supporting the ongoing uncertainty regarding its clinical utility.

A key strength of this study is the inclusion of a broad clinical spectrum of invasive listeriosis across 4 decades, encompassing bloodstream infection, CNS disease, endovascular infection, and other focal manifestations. This single-center US cohort provides a pragmatic perspective on diagnostic challenges, treatment patterns, and both short- and long-term outcomes.

However, several limitations merit consideration. First, this retrospective single-center study may limit generalizability. The relatively small sample size and limited number of mortality events restrict statistical power and increase risk of model overfitting, Analyses were primarily univariate. Residual confounding by disease severity or other factors cannot be excluded. Neurological outcomes were captured as a binary variable without systematic documentation of the type or severity of deficits, limiting comparisons across patients.

Second, missing data and variability in diagnostic testing, including the later introduction of multiplex PCR panels, limited our ability to assess discordance between testing modalities. In addition, the timing of specimen collection (blood vs CSF) was not consistently available, precluding evaluation of the relative diagnostic performance of each test.

Third, treatment selection was nonrandomized, introducing potential confounding by disease severity, particularly in analyses assessing adjunctive therapy. Use of gentamicin varied over time, with treatment durations ranging from brief to prolonged courses, limiting assessment of dose or duration-dependent effects. Patients receiving adjunctive gentamicin may also have had more severe disease, introducing confounding by indication that could not be addressed in univariate analyses.

Finally, the long study period spans changes in diagnostic methods and antimicrobial practices that may have influenced outcomes. No consistent temporal improvement in mortality was observed across decades, although this analysis was limited by small sample sizes and was not powered to detect modest changes. Long-term all-cause mortality may also reflect underlying comorbidities or unrelated causes rather than infection-attributable outcomes.

In summary, outcomes in invasive listeriosis are largely driven by host factors and severity of illness at presentation. Blood cultures remain critical for the diagnosis of neurolisteriosis, and PCR may provide additional diagnostic utility when CSF cultures are negative. Aminopenicillin-based therapy remains the cornerstone of treatment, while adjunctive gentamicin was not associated with improved outcomes in this cohort. Larger multicenter prospective studies are needed to better define optimal antimicrobial strategies in this high-risk population.

## Supplementary Material

ofag496_Supplementary_Data

## Supplementary Data

Supplementary materials are available at *Open Forum Infectious Diseases* online. Consisting of data provided by the authors to benefit the reader, the posted materials are not copyedited and are the sole responsibility of the authors, so questions or comments should be addressed to the corresponding author.
