# Supplementary material for: Clinical Characteristics and Outcomes of Invasive Listeria Monocytogenes Infection: A 45-Year Retrospective Study at a US Tertiary Center
Source: Open Forum Infect Dis. 2026 Aug 12;13(9):ofag496. doi: 10.1093/ofid/ofag496 (PMC13589291; doi:10.1093/ofid/ofag496)
Supplement: Supplementary file 1 [file ofag496_supplementary_data.docx]

**Supplementary material:**

**Supplementary Figure 1**

S figure 1: Kaplan–Meier estimates of 90‑day survival among patients with invasive listeriosis stratified by receipt of penicillin‑based therapy. Survival curves were compared using the log‑rank test.

**Supplementary Table 1: Subjective exposure details (if any)**

| Exposure Category | Specific Examples | Number of Patients* (27) |
| --- | --- | --- |
| Dairy products | Unpasteurized milk/cheese, soft cheeses (blue, brie), cheesecake, coffee creamer | 8 |
| Meat / seafood | Frozen foods/deli items, undercooked meat, hot dogs (Arizona outbreak 1999), raw fish, wild bear meat | 14 |
| Fast food / processed food | Fast food, fried chicken | 3 |
| Environmental / activity-related | Camping in Northern Minnesota, island travel, extensive airplane travel, hay exposure, plumbing work | 5 |

*Not mutually exclusive categories

**Supplementary Table 2: Neurolisteriosis: clinical characteristics and management**

| Neurolisteriosis | Patients (n=18) |
| --- | --- |
| Gender (Male) | 9 (50%) |
| Age [Median, IQR] | 60.5 years [IQR: 44.5-69.5] |
| Immunocompromised | 11 (61.1%) |
| CSF analysis (median, IQR) |  |
| CSF protein | 162 mg/dl (IQR: 106.0-286.5) |
| CSF glucose | 42 mg/dl (IQR:29.8-81.3) |
| CSF white cell count | 322.5 cells/mm3 (IQR: 118.3-1643.5). |
| CSF culture positivity | 57.1% (n=8/14) |
| CSF PCR positivity | 71.4 % (n=5/7) |
| Imaging |  |
| Rhombencephalitis | 41.6% (n=5/12) |
| Treatment |  |
| Ampicillin plus gentamicin | 38.9% (n=7) |
| ampicillin monotherapy | 22.2% (n=4) |
| Steroid continuation or use | 44.4% (n=8) |
| Outcomes |  |
| Residual neurological deficits | 8 (44.4%) |

**Supplementary Table 5:**

| Study | Design / Setting | Neurolisteriosis Cohort | Mortality, n (%) (Timepoint) | Neurologic Sequelae | Treatment Signal |
| --- | --- | --- | --- | --- | --- |
| Arslan et al., 2015 | Multinational retrospective study | Neuroinvasive listeriosis | ~20–40% (in-hospital) | Frequent residual deficits | No definitive treatment superiority demonstrated |
| Thønnings et al., 2016 | Nationwide retrospective cohort (Denmark) | CNS infection and bacteremia | ~25–30% (30-day) | Not well described | No clear mortality benefit with aminoglycoside combination therapy |
| Charlier et al., 2017 (MONALISA) | Prospective national cohort (France) | ~250 patients with neurolisteriosis | ~30% (3-month) | Common; not systematically quantified | Aminoglycoside use associated with improved survival; dexamethasone associated with increased mortality |
| Dickstein et al., 2019 | Multicenter retrospective cohort | Invasive listeriosis (CNS subgroup included) | ~20–30% (30-day) | Limited data | No consistent benefit of aminoglycoside therapy |
| Sutter et al., 2024 | Retrospective cohort (Germany) | Invasive listeriosis (including CNS cases) | ~20–30% (in-hospital) | Not a primary outcome | Gentamicin combination associated with lower mortality |
| Villa et al., 2025 | Multicenter ICU cohort | Severe listeriosis (high CNS burden) | >30–40% (ICU/in-hospital) | Not systematically reported | Outcomes driven primarily by severity of illness rather than antimicrobial regimen |

**Supplementary Table 4:** Logistic regression analysis for neurological deficits among neurolisteriosis

| Variable | Neurological deficits, No (N=10) | Neurological deficits, yes (N=8) | P value |
| --- | --- | --- | --- |
| **Age at diagnosis**  **- Median (Q1, Q3)** | **47 (27.5, 61)** | **67.5(60.75,72)** | **0.033** |
| CSF glucose | 42 (30.25, 80) | 43.5 (29.25, 69.25) | 1.0 |
| CSF Protein | 159.5 (106, 295.5) | 162 (123.25, 216) | 0.67 |
| CSF white cell count | 1307 (337, 2088.75) | 122.5 (85.9, 200) | 0.088 |
| Days from symptom onset to diagnosis | 2 (2, 3) | 4.5 (2, 9) | 0.151 |
| Gender male | 6 (60%) | 3 (37.5%) | 0.343 |
| Immunocompromised | 7 (70.0%) | 4 (50%) | 0.387 |
| Steroid use | 5 (50%) | 3 (37.5%) | 0.596 |
| **ICU admission** | **3 (30.0%)** | **6 (75.0%)** | **0.058** |
| Gentamicin use | 3 (30%) | 4 (50%) | 0.387 |

Data presented as n (%) or mean ± SD and median (Q1–Q3) for continuous variables. values calculated using Pearson’s chi-square test for categorical variables and Kruskal–Walli’s test for continuous variables. AMS = altered mental status; ICU = intensive care unit; CNS = central nervous system

Supplementary table 3: Clinical characteristics, management, and outcomes of pregnancy-associated invasive listeriosis cases (n = 2)

| Maternal age (years) | Gestational age (weeks) | Clinical presentation | Diagnostic test | Treatment | Outcome |
| --- | --- | --- | --- | --- | --- |
| 28 | 27 | Chills, precipitous  labor | Placental  culture | No maternal antimicrobial therapy; neonate received gentamicin for 2 weeks | Neonatal  death at 5  weeks (cardiac  complications) |
| 24 | 38 | Nausea, vomiting, fever,  meningeal  signs | CSF PCR | IV  Ampicillin for 3  weeks plus IV  Gentamicin for  1 week | Survived without  complications |

Table describes maternal pregnancy-associated cases and neonatal outcomes when available for contextual clinical reporting; neonates were not part of the analytic cohort.

SUMMARY: In a 45-year cohort of invasive listeriosis, 1-year mortality was 20% and was associated with immunocompromised status and ICU admission, while adjunctive gentamicin was not associated with improved survival.

SUPPLEMENTARY MATERIAL SUPPLEMENTARY
